# Comparative Study of Elastic Network Model and Protein Contact Network for Protein Complexes: The Hemoglobin Case

**DOI:** 10.1155/2017/2483264

**Published:** 2017-01-22

**Authors:** Guang Hu, Luisa Di Paola, Zhongjie Liang, Alessandro Giuliani

**Affiliations:** ^1^Center for Systems Biology, School of Electronic and Information Engineering, Soochow University, Suzhou 215006, China; ^2^Unit of Chemical-Physics Fundamentals in Chemical Engineering, Department of Engineering, Università Campus Bio-Medico di Roma, Rome, Italy; ^3^Environment and Health Department, Istituto Superiore di Sanità, Viale Regina Elena 299, 00161 Roma, Italy

## Abstract

The overall topology and interfacial interactions play key roles in understanding structural and functional principles of protein complexes. Elastic Network Model (ENM) and Protein Contact Network (PCN) are two widely used methods for high throughput investigation of structures and interactions within protein complexes. In this work, the comparative analysis of ENM and PCN relative to hemoglobin (Hb) was taken as case study. We examine four types of structural and dynamical paradigms, namely, conformational change between different states of Hbs, modular analysis, allosteric mechanisms studies, and interface characterization of an Hb. The comparative study shows that ENM has an advantage in studying dynamical properties and protein-protein interfaces, while PCN is better for describing protein structures quantitatively both from local and from global levels. We suggest that the integration of ENM and PCN would give a potential but powerful tool in structural systems biology.

## 1. Introduction

Proteins rarely act alone: in the great majority of cases they perform a vast array of biological functions by forming functional complexes [1, 2]. The study of protein complexes not only elucidates the molecular mechanism of many diseases [3] but also provides structural information of protein-protein interactions [4]. With the increasing number of structural data, a lot of regularities have been found for protein complexes based on their topological structures [5]. However, the structural and assembly principles underlying protein complexes organization are not yet fully understood, which poses a great challenge in structural systems biology [6]. A well-studied example of protein complex is hemoglobin (Hb) tetramer, which contains two *α* and two *β* subunits as a dimer of dimer [7]. Hbs exist in three quaternary conformations: the low-affinity (deoxy, *T*) state and the high-affinity (oxy, *R*; carbonmonoxy, *R*2) states. Hbs are never present in cells as monomers. Therefore, Hbs were considered as a sort of ‘obliged' allosteric protein complexes and, even thanks to the great amount of both structural and physiological data, attracted a lot of attentions [8–10].

Network theory has become a versatile method to study structures and dynamics of biological systems [11–13]. As a dynamical-based method introduced by Tirion [14], Elastic Network Model (ENM) allows performing normal mode analysis at *C*_*α*_ network level. Two mostly used ENM methods, Gaussian Network Model (GNM) and Anisotropic Network Model (ANM), were further proposed by Bahar and coworkers [15, 16]. ENM is an efficient computational tool to describe the essential vibrational dynamics encoded in the molecular topology [17–20]. It has been proved that the low-frequency modes of ENM are critical of collective motions [21], while the high-frequency modes can identify hot spots for protein-protein interactions [22].

The approach of Protein Contact Network (PCN) was proposed by Kannan and Vishveshwara [23] and now has become a new paradigm in protein ontology [24–28]. In a PCN, nodes correspond to *C*_*α*_, while edges exist if two amino acid residues (nodes) are close to each other under different cutoffs [29]. Based on this graphical representation, different topological parameters have been developed to describe protein structures and functions from both the global and the local prospective [30–32].

Both ENM and PCN offer computationally efficient tools to study the structure and function of protein complexes [33, 34], from predicting functionally important residues [35, 36], to characterize protein-protein interactions [37, 38] and allosteric communication paths [39, 40]. Of course, both models have strengths and weaknesses and their comparative study is needed.

In this paper, we have analyzed and compared four applications of ENM and PCN on Hb structures: conformational change characterization, modular analysis, allosteric mechanisms investigation, and interface characterization. Although there are several works reported on the ENM [41–43] and PCN [44, 45] studies of Hb independently, this work revisits Hb as case study and mainly focuses on the methodology comparison of ENM (specifically GNM and ANM) and PCN.

## 2. Materials and Methods

### 2.1. Data Sets

Hemoglobins (Hbs) have three states [7]. We select their structures for the ENM and PCN analysis, which are listed as follows: *T*-Hb (PDB code: 2dn2), *R*-Hb (PDB code: 2dn1), and *R*2-Hb (PDB code: 2dn3).

### 2.2. Gaussian Network Model and Anisotropic Network Model

GNM [15] describes a protein as a network of *C*_*α*_ connected by springs of uniform force constant *γ* if they are located within a cutoff distance *r*_*c*_ (7 Å in this study). In GNM, the interaction potential for a protein of *N* residues is [46]

(1) $$\begin{array}{c} V_{GNM}=-\frac{\gamma }{2}\left[\;\overset{N-1}{\underset{i=1}{\sum }}\overset{N}{\underset{j=i+1}{\sum }}\left(\;R_{ij}-R_{ij}^{0}\;\right)\cdot \left(\;R_{ij}-R_{ij}^{0}\;\right)\Gamma_{ij}\;\right], \end{array}$$

where *R*_*ij*_ and *R*_*ij*_^0^ are the equilibrium and instantaneous distance between residues *i* and *j*, and *Г* is *N* × *N Kirchhoff matrix*, which is written as follows:

(2) $$\begin{array}{c} \Gamma_{ij}=\left\{\begin{array}{cc} -1 & i\neq j,\;\;R_{ij}\leq r_{c}\\ 0 & i\neq j,\;\;R_{ij}\geq r_{c}\\ -\underset{i,i\neq j}{\sum }\Gamma_{ij} & i=j. \end{array}\right. \end{array}$$

Then, square fluctuations are given by

(3) ΔRi2=3kTγ·Γ−1ii,ΔRi·ΔRj=3kTγ·Γ−1ij.

The normal modes are extracted by eigenvalue decomposition: Γ = *U*Λ*U*^*T*^, where *U* is the orthogonal matrix whose *k*th column *u*_*k*_ is *k*th mode eigenvector. Λ is the diagonal matrix of eigenvalues, *λ*_*k*_. 〈Δ*R*_*i*_ · Δ*R*_*j*_〉 can be written in terms of the sum of the contribution of each mode as follows:

(4) $$\begin{array}{c} \langle \;\Delta R_{i}\cdot \Delta R_{j}\;\rangle =\left(\;\frac{3kT}{\gamma }\;\right)\cdot \underset{k}{\sum }{\left[\;{\left(\;U_{k}\Lambda_{k}U_{k}^{T}\;\right)}^{-1}\;\right]}_{ij}. \end{array}$$

Thus, the cross-correlation can be calculated by

(5) $$\begin{array}{c} C_{ij}=\frac{\langle \Delta R_{i}\cdot \Delta R_{j}\rangle }{{\left[{\langle \Delta R_{i}\rangle }^{2}\cdot {\langle \Delta R_{j}\rangle }^{2}\right]}^{1/2}}. \end{array}$$

The cross-correlation value ranges from −1 to 1: positive values mean that two residues have correlated motions, while the negative values mean that they have anticorrelated motions.

In ANM [16], the interaction potential for a protein of *N* residues is [46]

(6) $$\begin{array}{c} V_{ANM}=-\frac{\gamma }{2}\left[\;\overset{N-1}{\underset{i=1}{\sum }}\overset{N}{\underset{j=i+1}{\sum }}{\left(\;R_{ij}-R_{ij}^{0}\;\right)}^{2}\Gamma_{ij}\;\right]. \end{array}$$

The motion of the ANM mode of proteins is determined by 3*N* × 3*N Hessian matrix H*, whose generic element is given as follows:

(7) $$\begin{array}{c} H_{ij}=\left[\begin{array}{ccc} \frac{\partial^{2}V}{\partial X_{i}\partial X_{j}} & \frac{\partial^{2}V}{\partial X_{i}\partial Y_{j}} & \frac{\partial^{2}V}{\partial X_{i}\partial Z_{j}}\\ \frac{\partial^{2}V}{\partial Y_{i}\partial X_{j}} & \frac{\partial^{2}V}{\partial Y_{i}\partial Y_{j}} & \frac{\partial^{2}V}{\partial Y_{i}\partial Z_{j}}\\ \frac{\partial^{2}V}{\partial Z_{i}\partial X_{j}} & \frac{\partial^{2}V}{\partial Z_{i}\partial Y_{j}} & \frac{\partial^{2}V}{\partial Z_{i}\partial Z_{j}} \end{array}\right], \end{array}$$

where *X*_*i*_, *Y*_*i*_, and *Z*_*i*_ represent the Cartesian components of residues *i* and *V* is the potential energy of the system. *r*_*c*_ used here is 13 Å. Accordingly, ANMs provide the information not only about the amplitudes but also about the direction of residue fluctuations.

The similarity between two ANM modes, *u*_*k*_ and *v*_*l*_, evaluated for proteins with two different conformations can be quantified in terms of inner product of their eigenvectors [39]; that is,

(8) $$\begin{array}{c} O\left(\;u_{k},v_{l}\;\right)=u_{k}\cdot v_{l}. \end{array}$$

The degree of overlap between *k*th ANM modes *u*_*k*_ and the experimentally observed conformation change Δ*r* of Hbs among different states is quantified by ((Δ*r* · *u*_*k*_)/|Δ*r*|). Therefore, the cumulative overlap CO(*m*) between Δ*r* and the directions spanned by subsets of *m* ANM modes is calculated as follows:

(9) $$\begin{array}{c} CO\left(\;m\;\right)=\sqrt{\overset{m}{\underset{k=1}{\sum }}{\left(\Delta r\cdot \frac{u_{k}}{\left|\Delta r\right|}\right)}^{2}}. \end{array}$$

The Markov model coupled with GNM was used for exploring the signal transductions of perturbations in proteins [47, 48]. The affinity matrix *A* describes the interactions between residue pairs connected in GNM; its generic element *a*_*ij*_ is defined as follows:

(10) $$\begin{array}{c} a_{ij}=\frac{N_{ij}}{\sqrt{N_{i}N_{j}}}, \end{array}$$

where *N*_*ij*_ is the number of atom-atom contacts between residues *i* and *j* based on a cutoff distance of 4 Å and *N*_*i*_ is the number of side-chain atoms in residue *i*. The density of contacts at each node *i* is given by

(11) $$\begin{array}{c} d_{i}=\overset{N}{\underset{j=1}{\sum }}a_{ij}. \end{array}$$

The Markov transition matrix *M*, whose element *m*_*ij*_ = *d*_*j*_^−1^*a*_*ij*_, determines the conditional probability of transmitting a signal from residue *j* to residue *i* in one time step. Accordingly, the hitting time for the transfer of a signal from residue *j* to *i* is given by [47]

(12) Hi,j=∑k=1NΓ−1kj−Γ−1ij−Γ−1ki−Γ−1ii·dk,

where *Г* is Kirchhoff matrix obtained by GNM. The average hit time for *i*th residue 〈*H*(*i*)〉 is the average of *H*(*i*, *j*) over all starting points *i*. The commute time is defined by the sum of the hitting times in both directions; that is,

(13) $$\begin{array}{c} C\left(\;i,j\;\right)=H\left(\;i,j\;\right)+H\left(\;j,i\;\right). \end{array}$$

*C*(*i*, *j*) was defined as the corresponding distance, as the weight of the edge between node *i* and *j* in the network.

### 2.3. Protein Contact Networks (PCNs)

Protein Contact Networks (PCNs) provide a coarse-grained representation of protein structure [49], based on *Cα* coordinates from PDB files: network nodes are the residues, while links exist between nodes whose Euclidean distance (computed with respect to *α*-carbons) is within 4 to 8 Å, in order to account only for significant noncovalent intramolecular interactions [24, 50, 51].

After building up the network, it is possible to quantify its features through the adjacency matrix Ad, whose generic element Ad_*ij*_ is 1 if *i*th and *j*th nodes are connected by a link; otherwise it is 0.

The most basic descriptor is the* node degree*, defined for each node as the number of links involving the node itself:

(14) $$\begin{array}{c} k_{i}=\sum_{j}{Ad}_{ij}. \end{array}$$

**Figure 1:**
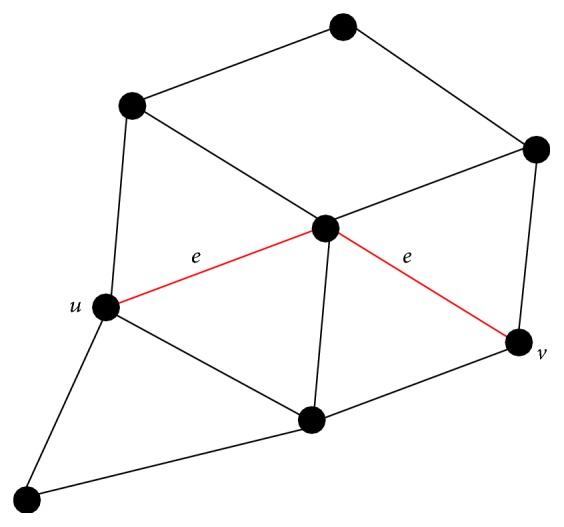
Example of a graph with 8 vertices and 13 edges, while the red line shows a path from vertices *u* to *v*.

Given a set of vertices *V*, the* shortest path *sp_*u*,*v*_ between two nodes *u*, *v* ∈ *V* is the minimum number of edges connecting them (Figure 1). Its role is crucial since it has been demonstrated that the lower the network* average shortest path* (or* characteristic length*, computed as the average value over the whole number of node pairs), the higher the efficiency of signal transmission through the network [52]. In PCNs the average shortest path describes the protein attitude to allosteric regulation.

The* betweenness centrality* of a node describes the number of shortest paths passing by it. Given a set of vertices *V*, the betweenness centrality of node *s* ∈ *V* is defined as follows:

(15) $$\begin{array}{c} betw\left(\;i\;\right)=\underset{v\in V,v\neq i}{\sum }\;\underset{u\in V,u\neq i}{\sum }\frac{\sigma_{v,u}\left(i\right)}{\sigma_{v,u}}, \end{array}$$

where *σ*_*v*,*u*_ is the total number of the shortest paths connecting two nodes *u*, *v* ∈ *V*, whereas *σ*_*v*,*u*_(*i*) represents the number of shortest paths connecting the nodes *u* and *v* passing on *i* as well. Therefore, high betweenness centrality nodes take part in many shortest paths, so their removal is likely to be noxious for the whole network connectivity. We computed the betweenness centrality by means of the algorithm described in [53].

Closeness centrality describes the general closeness of a node to all other nodes, in terms of length of shortest paths:

(16) $$\begin{array}{c} close\left(\;i\;\right)=\frac{1}{\sum_{u\in V,u\neq i}sp\left(u,i\right)}. \end{array}$$

Closeness centrality of residues in PCNs has been demonstrated to describe conformational transitions occurring in protein response to environmental stimuli through cooperative processes [54]: residues in the active site of enzymes show both high degree and closeness centrality; however, it does not provide any clue about allosteric regulation in the enzyme-substrate binding.

The Guimerà-Amaral cartography [55] provides a framework to classify nodes according to their topological role in the network. It is based on network clustering into nodes groups (clusters). We applied a spectral clustering procedure, previously demonstrated to catch functional modules in protein structures [56].

The spectral clustering algorithm [57] applies to the Laplacian matrix *L* defined as the difference between the adjacency matrix Ad and the degree matrix *D* (a diagonal matrix whose generic element *D*_*ii*_ is *i*th node degree). We applied the eigenvalue decomposition to *L*: the spectral clustering decomposition refers to the eigenvector *v*_2_ corresponding to the second minor eigenvalue.

The procedure applies iteratively to get the final desired number of clusters (set by defining the number of iterations); nodes are parted according to the sign of corresponding *v*_2_ components. So, for instance, if it is required to part the network into four clusters, the first partition produces two clusters, whose *v*_2_ components have opposite signs and, successively, both clusters undergo the same procedure, applied to single cluster nodes subset.

We represented the clustering partition in two ways: first, we reported on the ribbon representation residues pertaining to different clusters in different colors, to identify at once clusters on the three-dimensional structure representation. Second, we reported the clustering color map, a matrix whose generic element is colored not in blue if residues corresponding to indices pertain to the same cluster and in blue, background, if corresponding residues do not belong to the same cluster. This representation helps understanding the distribution of clusters along sequence.

After clustering partition, it is possible to compute for each node (residue) the* participation coefficient P*, defined as follows:

(17) $$\begin{array}{c} P_{i}=1-{\left(\;\frac{k_{si}}{k_{i}}\;\right)}^{2}. \end{array}$$

*k*_*i*_ is the overall degree of the node, *k*_*si*_ is the node degree in its own cluster (number of links the node is involved into with nodes pertaining to its own cluster).

A complementary descriptor is the intramodule connectivity *z*-score *z*, defined as follows:

(18) $$\begin{array}{c} z_{i}=\frac{k_{si}-{\overset{-}{k}}_{si}}{SD_{si}}, \end{array}$$

where $\overset{-}{k}$ and SD are the average value and the standard deviation of the degree *k* extended to the whole network. The descriptor *z* catches the attitude of nodes to preferentially connect with nodes in their own clusters; *z* strongly correlates with node degree, so high *z* residues are mostly responsible for global protein stability.

The participation coefficient *P* has been previously demonstrated of a crucial importance in identifying key residues in protein structure with a functional role [43, 56, 58]; residues with *P* values higher than 0.75 are mostly devoted to the communication between modules (clusters), since they spend more than half of their links with residues pertaining to clusters other than theirs. In other words, signaling pathways between clusters pass by them.

*P*-*z* maps show a peculiar shape (“dentist's chair”) for PCNs [58]: high *P* residues show low *z* values, meaning the role of nodes (communication, high *P*, and *z*) are well separated. We previously reported [35] that in protein-ligand binding *P* shifts from nonnull to null values for residues close to an active site in allosteric proteins.

We computed for each structure *P* profile and *P*-*z* maps. Then, for the two pairs apo-holo forms we report the heat maps of *P* variation on the ribbon structure, so to highlight regions in the protein structure undergoing changes upon ligand binding.

The analysis was performed by means of a purposed software implemented in Matlab environment v 2014a, including functions from Bioinformatics Toolbox. Heat maps of *P* variation (comparison between holo and apo forms), Guimerà-Amaral cartography and clusters onto the protein ribbon representation, have been produced by means of a purposed Python script compiled in the embedded Python environment; for further details and application of the method see [37].

Table 1 sums up PCN descriptors, along with their structural and biological relevance.

## 3. Results and Discussion

### 3.1. ENM Results

GNM and ANM are simple yet effective methods [33]. GNM can only describe the amplitude of residue fluctuations, but ANM can give the direction of the motions. In this section, ANM was used to investigate conformational change between *T*-Hb and *R*-Hb, and describe the dynamical properties of protein-protein interfaces. GNM was employed for the modular analysis of Hbs, which was coupled with Markovian stochastic analysis to study the allosteric mechanisms of Hbs. Expecting for the conformational change, we only chose *T*-Hb to exhibit these investigations. ENM results for other two states of Hbs show similar results, as shown in the supporting information.

#### 3.1.1. Conformational Change

**Figure 2:**
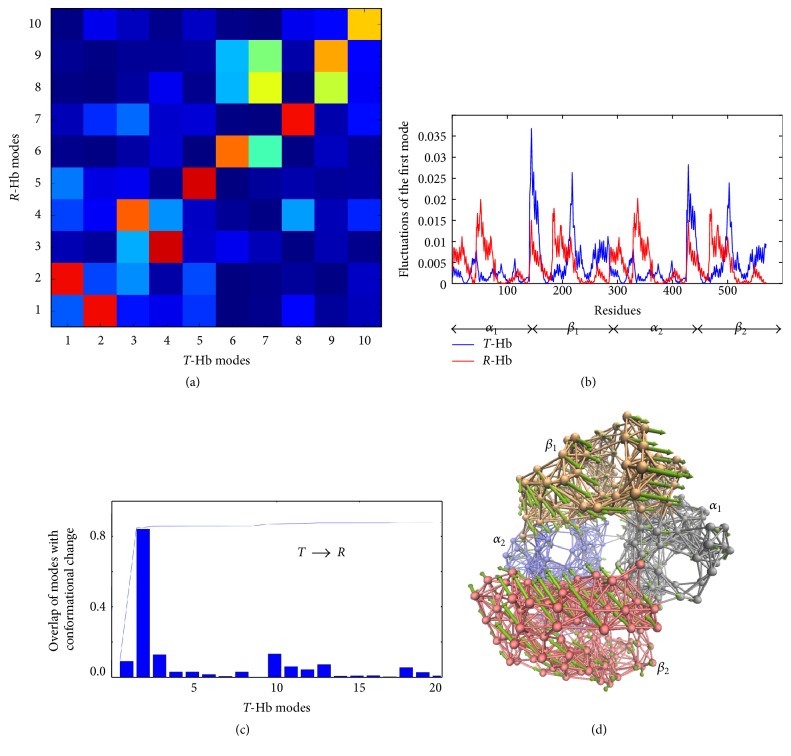
ENM results for *T* → *R* transition of tetrameric Hb. (a) Overlaps between the ten slowest ANM modes of *T*- and *R*-Hbs. (b) Distribution of mean-square fluctuations obtained by the first ANM mode of *T*- and *R*-Hbs. The residue index of the four chains is 1–140 (*α*_1_), 141–285 (*β*_1_), 286–425 (*α*_2_), and 426–570 (*β*_2_). (c) Overlaps of individual *T*-Hb ANM modes with the conformational change within *T* → *R* transition. (d) The motion of the second ANM mode of *T*-Hb; here the protein is represented as a network.

ENM results for the transition of tetrameric Hb between *T*-state (PDB code: 2dn2) and *R*-state (PDB code: 2dn1) are shown in Figure 2, while the results of the conformational change between *T*-state and *R*2-state (PDB code: 2dn3) and *R*-state and *R*2-state are shown in Supplementary Figure S1 in Supplementary Material available online at https://doi.org/10.1155/2017/2483264. First, the overlap map between the ten ANM slowest modes was calculated to compare the global dynamics of *T*- and *R*-Hbs. Along the diagonal in Figure 2(a), only the fifth and sixth modes are maintained, with the overlap values of 0.92 and 0.79. For other global modes, there are weaker correlations between two conformations. For example, the reordering of the first two modes was found, which means that the motion of the first mode of *T*-Hb is similar to the motion of the second mode of *R*-Hb, while the first mode of *R*-Hb shifts to the second mode of *T*-Hb. This result shows that global dynamics greatly changes between the two different states, even for the lowest mode. Then, the difference of two states was further investigated by the distribution of mean-square fluctuations driven by their global ANM modes, as shown in Figure 2(b). For the first mode *T*-Hb, the two *α* chains exhibit different dynamical behavior with two *β* chains, but two dimers of *α*_1_*β*_1_ and *α*_2_*β*_2_ show similar global dynamics (the blue line). For the first mode of *R*-Hb, the mean-square fluctuations profile of *α*-chain is very similar to that of the *β*-chain (the red line). Comparing these two structures the fact that *α* chains are more stable and *β* chains are less stable in *T* state than in *R* state emerges.

Overlaps of each ANM mode with the structural difference between *T* and *R* conformations were calculated to detect which individual mode contributes significantly to the structural differences between the results from experimental study and are calculated by (9).  Figure 2(c) shows that the transformation from *T* into *R* is favored by the second mode of *T*-Hb with the highest overlap (0.84). In this mode, the global motion involves quaternary changes of two dimers, namely, *α*_1_*β*_1_ dimer, exhibiting a torsional rotation in an opposite direction with *α*_2_*β*_2_ dimer (Figure 2(d)). Furthermore, this mode is also coordinated by hinge sites at *α*_1_-*β*_1_ and *α*_2_-*β*_2_ interface. Tekpinar and Zheng [42] have previously performed the ENM study of conformation changes from *T* to *R*_2_ structures, in which they found the first two modes contribute significantly to the conformational change. Our revisiting is in accordance with their results, because mode 2 observed herein seems like the combination motion of their two modes.

#### 3.1.2. Modular Analysis

**Figure 3:**
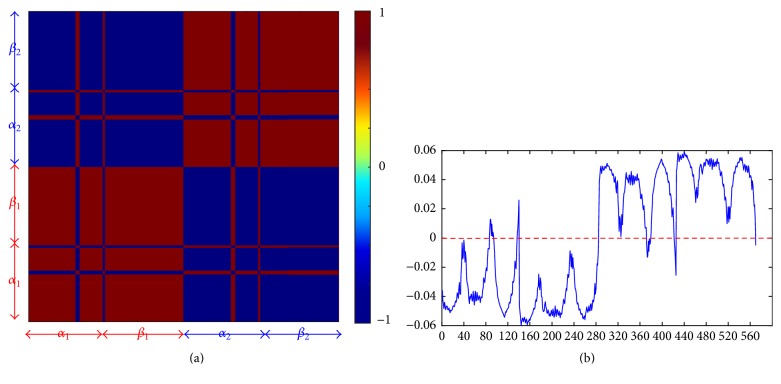
Modular analysis of *T*-Hb based on GNM. (a) The correlation map corresponding to the first mode divides the Hb into two modules. Red regions correspond to collective residue motions and blue-colored regions correspond to uncorrelated motions. (b) The shape of the first mode, which not only shows two modules but also predicts hinge sites.

In their recent work, Li et al. [59] developed a new method based on GNM and ANM for dividing a protein into intrinsic dynamics modular analysis. Here, we adopted a much simpler way, just based on the analysis of the GNM lowest mode. Correlation maps for cross-correlation not only describe collective motion but also reflect the symmetry of proteins [36]. To our aim, the correlation map for the first GNM mode was used for the modular analysis of Hb [60]. In the map, red indicates the highly correlated motions, blue represents the anticorrelated motions, and green is for the uncorrelated motions. As shown in Figure 3(a), the correlation map shows that *T*-Hb tetramer is divided into two modules, which correspond to *α*_1_*β*_1_ dimer and *α*_2_*β*_2_ dimer. Two red blocks indicate that *α*_1_ and *α*_2_ move in the same direction with *β*_1_ and *β*_2_, respectively. Blue blocks indicate that opposite motions are observed between these dimers.

Although the first GNM mode can only generate two modules, it can provide more dynamical information. After diagonalizing the Kirchhoff matrix, the first eigenvector corresponding to the highest eigenvalue can be derived and interpreted to represent the shape of a mode [61].  Figure 3(b) demonstrates that the shape corresponds to GNM mode of the Hb tetramer. It is easy to see that the shape of *α*_1_*β*_1_ dimer distributes under zero and *α*_2_*β*_2_ dimer above zero. Thus, the eigenvectors also partition the structure into two modules. In addition, some hinge sites were predicated at near zero positions, which are Thr41, Ala88, and Pro95 in Chain A, His146 in Chain B, Phe98, Leu105, and Ser138 in Chain C, and His2 and His146 in Chain D. Note that these hinge sites always locate at *α*_1_-*β*_2_ and *α*_2_-*β*_1_ interfaces. ENM results for modular analysis of *R*-Hb and *R*2-Hb are shown in Supplementary Figure S2.

#### 3.1.3. Allosteric Mechanisms

**Figure 4:**
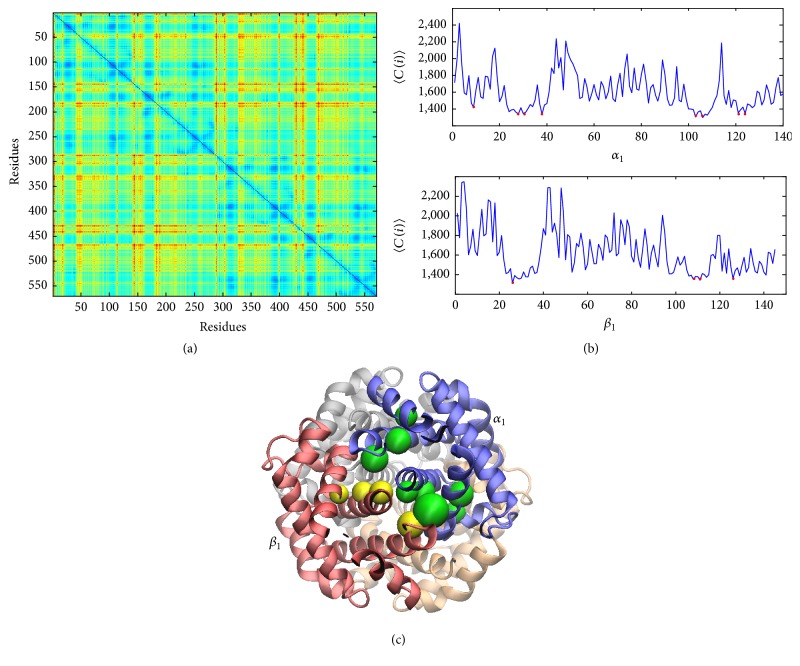
Signal propagation of residues for *T*-Hb. (a) The commute time map of *T*-Hb. Minimal of average commute time profiles (red circles) for *α*_1_ chain and *β*_1_ chain (b) indicates that most of residues with highest communication abilities (green beads in *α*_1_ chain and yellow beads in *β*_1_ chain) distribute at *α*_1_-*β*_1_ interface (c). Results are presented for 2dn1 (*T*), and 2dn2 (*R*) and 2dn3 (*R*_2_) showed similar behavior.

Communication inside protein complexes is implicit in collective motions which are inherent to the network topology [62]. Based on this idea, the signal-processing properties of residues can be investigated by Markovian stochastic analysis coupled with GNM [63, 64]. The commute time, *C*(*i*, *j*), a function of Markov transition probabilities, was used to measure the communication abilities of residue pairs.  Figure 4(a) displays the commute time map of *T*-Hb, while the blue and red regions correspond to short and long hit times. Furthermore, we calculated the average values of each row or column of the commute time map to evaluate the communication abilities of each residue. As shown in Figure 4(b), the minima of the average commute time 〈*C*(*i*)〉 indicate the key residues for *T*-Hb allostery. The profiles of average commute times for *α*_1_ chain and *β*_1_ chain indicate that Val10, Leu29, Arg31, Thr39, Cys104, Val107, His122, and Leu125 in *α*_1_ chain and Ala27, Val109, Cys112, and Gln127 in *β*_1_ chain are residues with highest communication abilities. It is worth mentioning that the two *α* chains and two *β* chains have the same profile shapes. The distributions of these residues in *α*_1_ chain and *β*_1_ chain are also displayed in the three-dimensional representation (Figure 4(c)). It was found that Arg31, Cys104, Val107, and His122 in *α*_1_ chain and Cys112 and Gln127 in *β*_1_ chain are located at *α*_1_-*β*_1_ interface. Likely, the same region was also found at *α*_2_-*β*_2_ interface. ENM results for modular analysis of *R*-Hb and *R*2-Hb are shown in Supplementary Figure S3.

#### 3.1.4. Interface Characterization

Protein interfaces are the sites where proteins or subunits physically interact. Identification and characterization of protein interfaces are not only important to understand the structures of protein complexes and protein-protein interactions, but also disease phenotypes [65]. Both GNM and ANM have been used to investigate protein-protein interfaces. Kantarci et al. [66] firstly applied GNM to classify interfaces of p53 core domain into the dimerization interface and crystal interface on the base of interfacial dynamics. Zen et al. [67] extended this method to study the interface of 22 representative dimers. More recently, Soner et al. [68] developed a web server to discriminate obligatory and nonobligatory protein complexes. Although GNM is the most used method to study protein-protein interfaces, we have showed here that ANM is also powerful to explore interfacial dynamics of Hbs.

Two kinds of interfaces have been classified in the Hb tetramer: allosteric sites located at *α*_1_-*β*_1_ and *α*_2_-*β*_2_ interfaces, which could be intended as allosteric interfaces. Hinge sites are detected always at *α*_1_-*β*_2_ and *α*_2_-*β*_1_ interfaces, providing structural interfaces. The analyses are in accordance with the results in Tekpinar and Zheng [42], which showed that allosteric interfaces are dynamically variable regions but not necessarily structural interfaces. In this section, square fluctuations of both monomeric and oligomeric proteins based on a large set of slow modes and the highest modes are compared for a deeper analysis of interfaces.

**Figure 5:**
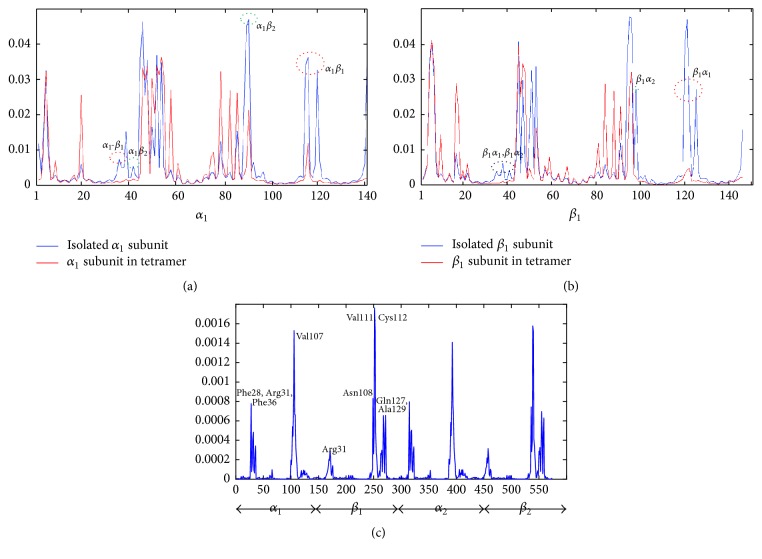
Square fluctuations of Hb monomers and tetramers. (a) Square fluctuations of *α*_1_ subunits in isolated and tetrameric states based on the first 20 ANM modes. The differences of mobilities at *α*_1_-*β*_1_ and *α*_1_-*β*_2_ interfaces are indicated by red and green circles. (b) Square fluctuations of *β*_1_ subunits in isolated and tetrameric states based on the first 20 ANM modes. Red, green, and black circles indicate the differences of mobilities at *α*_1_-*β*_1_ and *α*_2_-*β*_1_ interfaces and a common region of these two interfaces. (c) Square fluctuations of *T*-Hb tetramers based on the highest two modes. Hot spots are predicted by the peaks in the profile, while *α*_1_*β*_1_ and *α*_2_*β*_2_ dimer show the same prediction result.

Figures 5(a) and 5(b) show square fluctuations of *α*_1_ and *β*_1_ subunits in isolated and tetrameric states based on the first 20 ANM modes, while *α*_2_ and *β*_2_ subunits show similar behavior. Although *α* and *β* subunits are structurally identical, they are different in length and sequence. Accordingly, *α*_1_ and *β*_1_ subunits show different types of fluctuations, which have also been predicted by the previous molecular dynamics (MD) study [69]. The mobility of *α*_1_-*β*_1_ and *α*_1_-*β*_2_ interfacial residues of *α*_1_ subunit is reduced in the tetramer. The same happens for the mobilities of *α*_1_-*β*_1_ and *α*_2_-*β*_1_ interfacial residues of *β*_1_ subunit. Therefore, the flexibilities of residues located at both kinds of interfaces in bound states are lower than in unbound monomers. This kind of dynamical property of interfacial residues has also been detected by the MD simulation [70].

In addition, a similar region (residues 45–57) with high mobility in both isolated states was found, which corresponds to a long loop distributing between two adjacent subunits. The mobility of this region in *β*_1_ subunit was reduced, while no reduction was observed in *α*_1_ subunit. This may suggest that this long loop in *β* subunits is an allosteric region controlled by interfacial residues.

Among the interface residues, hotspots are defined as residues that have the greatest contribution to the binding energy. The prediction of hotspots is helpful not only to guide drug design but also to understand disease mutations [71]. Based on ENM results, Chennubhotla et al. [72] revealed that hot spots residues show a moderate-high flexibility at global modes. On the other hand, hot spots correlated very well with the residues with high mean-square fluctuations in the highest frequency modes in both GNM [73, 74] and ANM [22]. Ozbek et al. [74] have found that hot spots predictions based on the highest, the second and third highest, and the average three and five highest GNM modes show similar accuracies. Our calculation demonstrates that the square fluctuation based on two highest ANM modes is enough to predict the distribution of of Hb-tetramer hot spots. The result for *T*-Hb is shown in Figure 5(c). It is surprising that hot spots have been predicted only at *α*_1_-*β*_1_ and *α*_2_-*β*_2_ interfaces: Phe28, Arg31, Phe36, and Val107 in *α* subunits and Arg31, Asn108, Val111, Cys112, Gln127, and Ala129 in *β* subunits. Note that Arg31 and Val107 in *α* subunits and Arg31, Cys112, and Gln127 in *β* subunits are overlapped with allosteric sites. It also proved that allosteric interfaces rather than structural interfaces take part in the complex formation. The hotspots predicted for *R*- and *R*2-Hbs show small differences but still located at the same interfaces (Supplementary Figure S4).

### 3.2. PCN Results

**Figure 6:**
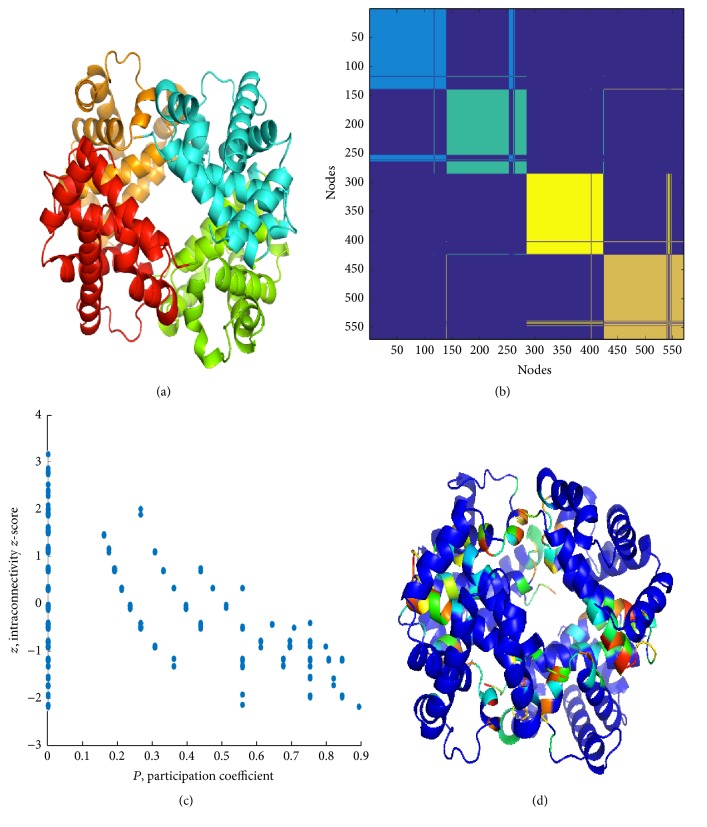
PCN results for *T*-Hb: (a) cluster partition, (b) cluster color map, (c) *P*-*z* map, and (d) *P* over ribbon. Cluster partition satisfactorily matches with chains, and high *P* residues are mostly located at the chain interfaces.

**Figure 7:**
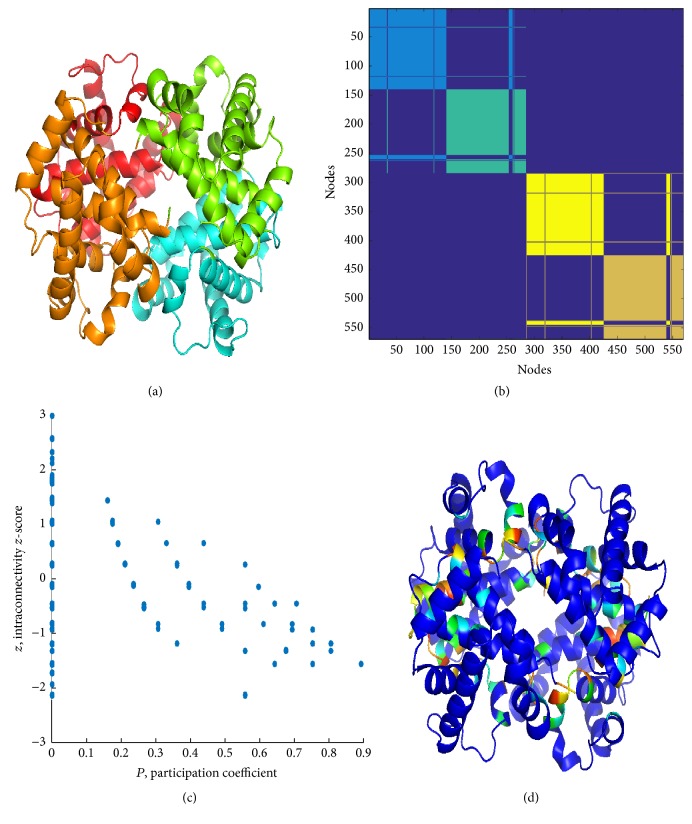
PCN results for *R*-Hb: (a) cluster partition, (b) cluster color map, (c) *P*-*z* map, and (d) *P* over ribbon. Again, clusters catch almost perfectly the chains and high *P* residues are located at the interfaces between chains. Similar results are obtained for *R*2-Hb; see Supplementary Figure S5.

**Figure 8:**
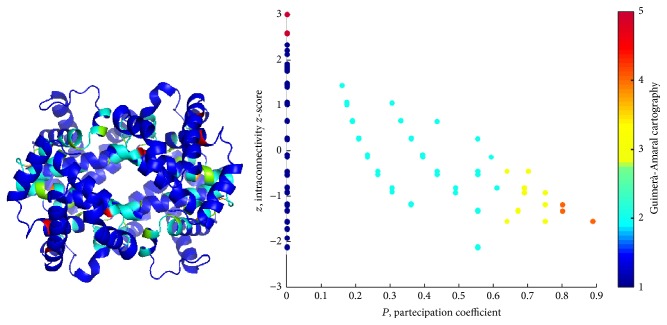
Guimerà-Amaral Cartography for *R*-Hb: only very few nodes are classified as hubs (*R*5) and located close to the active site. Non-hub kinless nodes (*R*4) are located in turn on the interface between chains and play a key role in the concerted motion underlying the allosteric regulation of hemoglobin.

**Figure 9:**
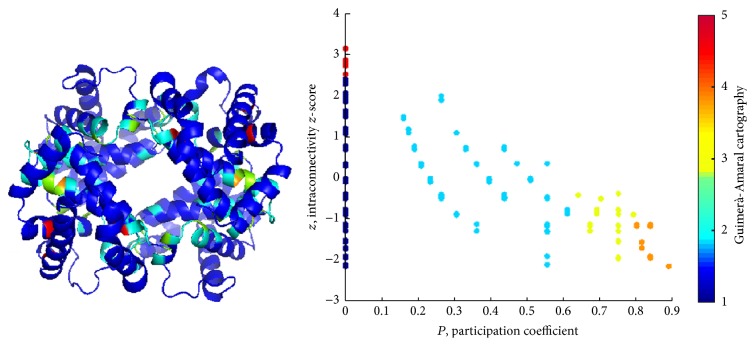
Guimerà-Amaral Cartography for *T*-Hb: very few nodes are classified as hubs (*R*5), but more than for *R*/O_2_ complex, again close to the active site. Non-hub kinless nodes (*R*4) lie on the interface between chains. Similar results hold for *R*2-Hb; see Supplementary Figure S6.

In this section, results from the application of PCN method and spectral clustering are reported for the three structures under enquiry. Figures 6 and 7 and Supplementary Figure S5 clearly show that cluster partition satisfactorily matches with chains, yet with some divergences (region pertaining to a chain falling in a cluster mainly composed of residues belonging to a second chain, “whiskers” in the clustering color map). In comparison with ENM, PCN results of three states of Hbs exhibit quite high similarity, even between *R*- and *R*2-states, as emerged mainly from the distribution of *P* along the ribbon structures (see Figures 8 and 9).

*Pz* maps show the typical profile for PCNs (and not for other real world networks), with most residues having *P* = 0 (only contacts with residues belonging to their own clusters). Residues with *P* > 0 are mostly interesting for protein functionality, since they account for signaling transmission through the protein structure (global property of protein structure).

High *P* residues are spotted in the structure and mostly (but not necessarily) placed in the interchain region. In previous works [35, 37, 51, 75], we demonstrated that the participation coefficient *P* addresses the functional role of residues in protein binding and, in general, identifies residues with a key role in protein structural and functional features.

*Pz* maps instruct a cartography, addressing a specific role to residues, as reported in Table 2. Hubs are nodes with *z* > 2.5, while *P* values address the role of nodes to connect different clusters. The Guimerà-Amaral cartography of the three Hbs is reported in Figures 8 and 9 and Supplementary Figure S6, as original form, on *Pz* maps, and projection on ribbon structures.

Noticeably, in PCNs *R*6 and *R*7 nodes are absent and only few *R*5 nodes are present, all at *P* = 0. In other words, high *z* nodes correspond to residues in charge for protein stability, while nonhub connector nodes are responsible for interdomain (intercluster) communication. Lys60 in *α*_1_, Glu26, His63, Lys66 in *β*_1_, and Leu28, Lys65, Leu68 in *β*_2_ in *T*-Hb, whereas Gly24, Lys61, and Leu141 in two *β* chains in *R*-Hb belong to *R*5 nodes. It is easy to note that *R*5 nodes distribute at *β* chains within the protein interior.

As previously stated [58], *R*4 nodes (nonhub kinless nodes) are crucial for the allosteric signal propagation: their kinless nature poses in the gray zone where residues acting at a global level play, so their role in the protein functionality is central. It was found that Leu91 and Arg92 in *α*_1_, His2, His116, and Ala129 in *β*_1_, His89 in *α*_2_, and Thr38, His116, and Phe118 in *T*-Hb and Trp37, Cys112, Phe122, and Pro124 in *R*-Hb belong to *R*5 nodes. Except His2, *R*4 residues were found at all four interfacial regions.

### 3.3. Comparison between ENM and PCN Results

We finally explicitly superpose the ENMs and PCNs results, in order to better specify key residues and features in allosteric regulation of Hb. Average commute times predict allosteric sites at both protein interior and two interfaces (*α*_1_-*β*_1_ and *α*_2_-*β*_2_ interfaces). In PCN, *R*4 nodes include allosteric sites at interfaces and *R*5 nodes include allosteric sites at protein interior. Combined with modular analysis and hot spots prediction, the use of ENM has advantage to classify protein-protein interfaces.

On the other hand, PCNs analysis relies upon a set of network descriptors to approach the study of protein structures quantitatively.  Table 3 reports the Pearson correlation coefficients between mean fluctuations and network descriptors, closeness centrality, betweenness centrality, and participation coefficient.

**Figure 10:**
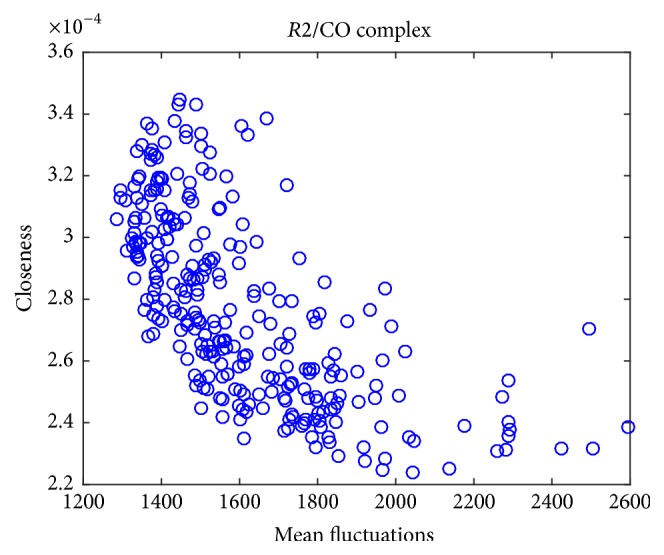
Closeness versus mean fluctuations: in this complex, there is an hyperbolic variation of closeness along with mean fluctuations: the closeness is thus a local rigidity descriptor.

Betweenness centrality poorly correlates (negatively) with mean fluctuations, while closeness anticorrelates more strongly with mean fluctuations, especially in the more rigid structure (*R*2/complex, Figure 10). The hyperbolic shape of the distribution confirms closeness is a general stiffness descriptor for protein structure. This property may indicate that closeness in PCN could provide an additional evidence to detect hinge sites. There is a relatively poor one-to-one correspondence of functional sites obtained between ENM and PCN, and thus the combination of these two approaches would improve the prediction.

## 4. Conclusion and Perspectives

ENM and PCN are light yet effective computational methods which simply require the three-dimensional coordinates of atoms in protein structures. In this work, the combination of the ENM and PCN methodologies has provided a plenty of information regarding the dynamic behavior of Hbs. It is noteworthy that the two classes of methods are able to catch the same features without a common, interexchange ground. In comparison with PCN, ENM can find the dominate motion for the conformational change of proteins and detect the dynamics of protein-protein interfaces observed by MD. Except for the topological parameters used in our work, there are more local and global network parameters that can be calculated in PCN to describe protein structures quantitatively. For example, residue centrality as a local network parameter was proposed to identify allosteric sites [76], and coefficient of assortativity as a global network parameter is related to the rates of protein folding [77]. In addition, we have found some correlations between ENM and PCN results. In previous studies [78], the average path lengths are highly correlated with residue fluctuations. Here, we show an additional positive correlation between residue fluctuations predicted by ENM and closeness centrality calculated by PCN. Although the general relationship between dynamical properties and more network parameters is needed to be established, we can conclude that ANM and GNM have advantages in studying dynamical properties and protein-protein interfaces, while PCN is better for describing structures quantitatively from both local and global levels.

In future, the combined description by means of these methods will largely contribute to understanding the dynamic behavior of complexes without heavy computational approaches, such as molecular dynamics (MD). Evidently, MD will anyway provide a very complete and fine description of dynamics, but the combination of lighter methods, such as ENM and PCN, will, for instance, guide MD simulations with well-grounded preliminary results, as preliminary approached in our previous works [79]. On the other hand, the two methods may help understanding the relationship between local fluctuation of residues and protein stability and functionality, being a primer for identifying key residues, responsible for lethal mutations. For example, the first attempt to combine ENM and PCN has been reported to investigate allosteric communication pathways [80]. In our work, we only indicate that ENM and PCN can be applied to four types of structural and dynamical paradigms. More detailed analysis for each case is needed. Although the integration of these two methods is just at the beginning, it would give a potential but powerful tool in structural systems biology.

## Supplementary Material

Figure S1 (a) shows that R2-Hb demonstrates different intrinsic dynamics with T-HB, and Figure S1 (b) detects that the second ANM mode of T-Hb contribute the most for the conformational change from T-state to R2 state. Figure S1 (c) shows that R2-Hb has similar intrinsic dynamics with R-Hb. Distribution of mean-square fluctuations shown in Figure S1 (d) further found that R2-Hb has similar intrinsic flexibilities with R-Hb, but different with T-Hb.

## Figures and Tables

**Table 1:** PCN descriptors and their structural and biological relevance.

PCN descriptor	Structural and biological role
Node degree *k*	Local stability [24]
Betweenness centrality (*betw*)	Signal transmission throughout the structure [26]
Closeness centrality (*close*)	Residues located in the active site of enzymes [26]
Participation Coefficient *P*	Signal transmission through modules (domains) [27]
Intramodule Connectivity *z*	Intramodule connectivity and communication [27]

**Table 2:** Guimerà-Amaral cartography.

	Regions	*z*	*P*
*Module nonhubs*	*R*1: ultraperipheral node	*z* < 2.5	*P* < 0.05
	*R*2: peripheral node	*z* < 2.5	0.05 < *P* < 0.625
	*R*3: nonhub connectors	*z* < 2.5	0.625 < *P* < 0.8
	*R*4: nonhub kinless nodes	*z* < 2.5	*P* > 0.8

*Module hubs*	*R*5: provincial hubs	*z* > 2.5	*P* < 0.3
	*R*6: connector hubs	*z* > 2.5	0.3 < *P* < 0.75
	*R*7: kinless hubs	*z* > 2.5	*P* > 0.75

**Table 3:** Pearson correlation coefficients of network descriptors with mean fluctuations (MF) and average commute times (CT).

	Closeness/MF	Betweenness/MF	P/MF	P/CT
2DN2	−0.3741	−0.1828	−0.0861	−0.2677
2DN1	−0.4320	−0.1226	−0.1878	−0.2904
2DN3	−0.7331	−0.1419	−0.3649	−0.2740
